# Case report: Sarcomatoid urothelial carcinoma of the renal pelvis masquerading as a renal abscess

**DOI:** 10.3389/fonc.2023.1055229

**Published:** 2023-01-23

**Authors:** Yaru Chu, Hao Ning, Ke Yin, Tong Chen, Haihu Wu, Delin Wang, Feifan Liu, Zhenlin Zhao, Jiaju Lv

**Affiliations:** ^1^ Department of Urology, Provincial Hospital Affiliated to Shandong First Medical University, Shandong First Medical University, Jinan, China; ^2^ Department of Urology, Shandong Provincial Hospital, Shandong University School of Medicine, Jinan, China; ^3^ Department of Pathology, Shandong Provincial Hospital, Shandong University School of Medicine, Jinan, China

**Keywords:** misdiagnosis, rare disease, renal pelvis, renal abscess, sarcomatoid urothelial carcinoma

## Abstract

Sarcomatoid urothelial carcinoma (SUC), a rare tumor of the urinary tract epithelium, exhibits a high degree of malignancy and therefore a poor prognosis. Due to the absence of specific clinical presentations and imaging findings, SUC of the renal pelvis masquerades as a renal abscess is frequently under-recognized or misdiagnosed as benign inflammatory disease, resulting in delayed or erroneous treatment. Here, we report a patient with SUC of the renal pelvis who presented with a renal abscess. Repeated anti-inflammatory treatment was ineffective. Unexpectedly, cancerous cells were detected in subsequent exfoliative cytology of nephrostomy drainage fluid. In accordance with this, radical surgery and postoperative chemotherapy were conducted. Fortunately, neither recurrence nor metastasis occurred during a one-year follow-up.

## Introduction

Renal pelvis carcinomas primarily arise in the form of transitional cell carcinomas arising from the urinary tract epithelium. SUC is a rare tumor type that accounts for just 0.3% of all urothelial carcinoma (UC) cases (1). SUCs are highly invasive, progress rapidly, and are generally associated with a very poor patient prognosis (2). Early diagnosis and appropriate treatment can improve prognosis (3). However, renal pelvis tumors may be mistaken for renal abscesses, leading to delayed diagnosis or misdiagnosis when specific clinical symptoms and imaging findings are lacking. SUCs of the renal pelvis masquerading as renal abscesses are extremely rare. In this report, we describe one such clinical case.

## Case report

**Figure 1:**
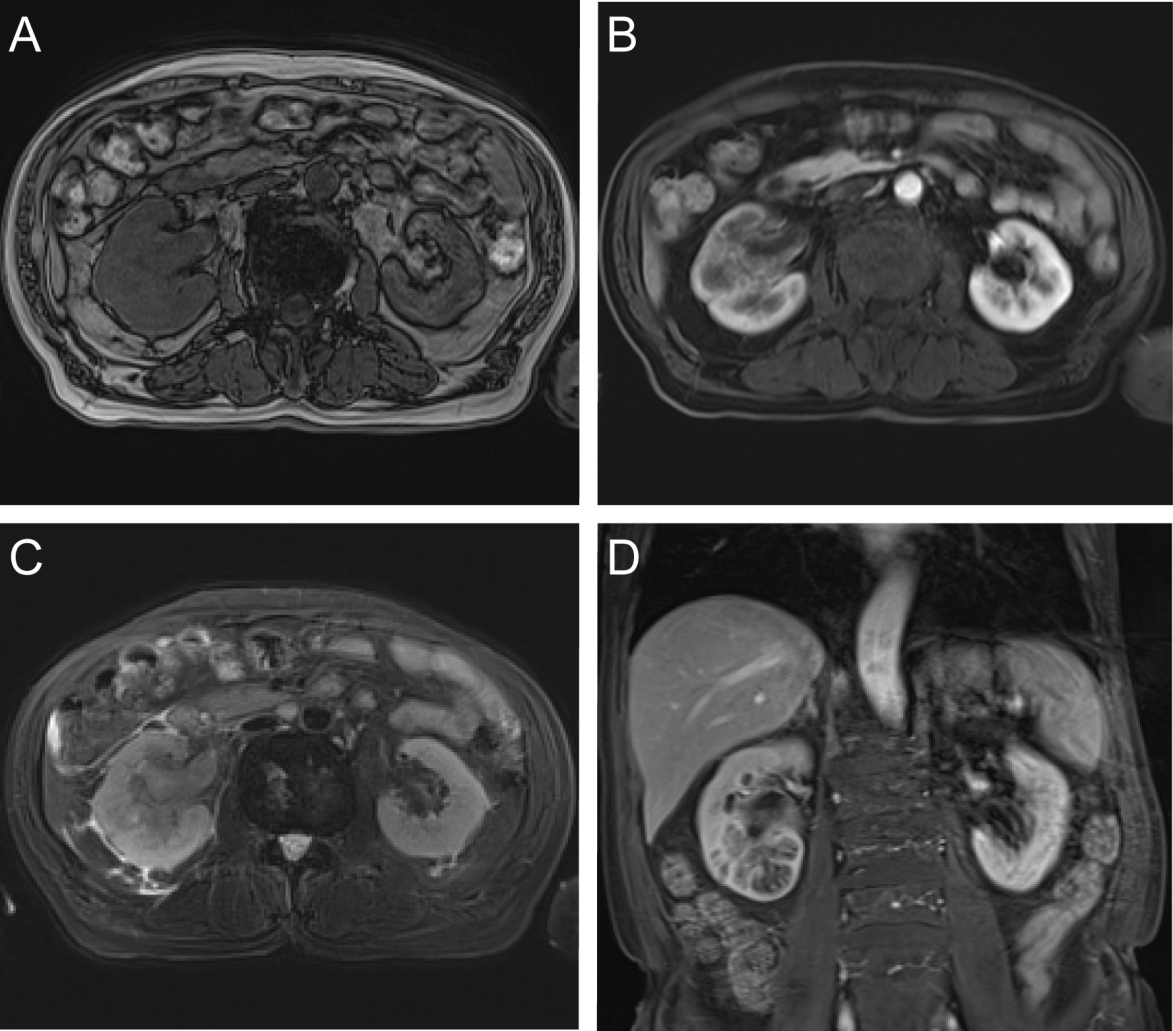
Enhanced MRI examination. **(A)** axial T1-weighted imging; **(B)** axial T1-weighted arterial phase imaging; **(C)** axial T2-weighted imging; **(D)** coronal T1-weighted arterial phase imaging.

**Figure 2:**
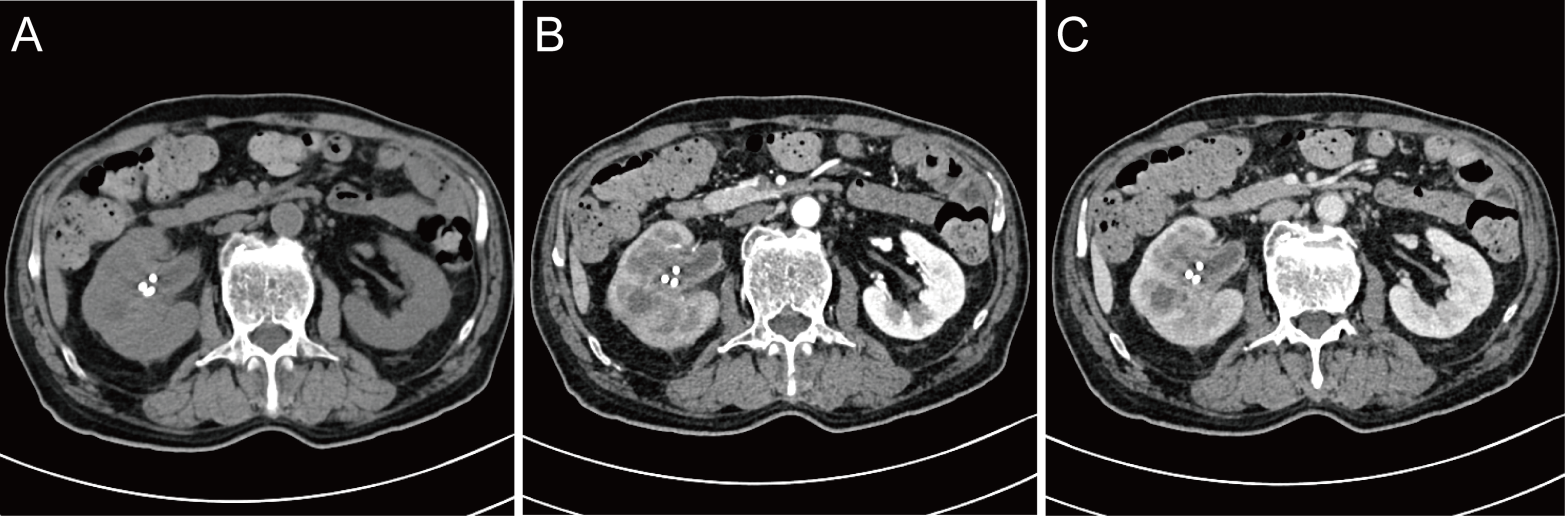
Review of enhanced CT after nephrostomy. **(A)** plain scan; **(B)** arterial phase; **(C)** venous phase.

**Figure 3:**
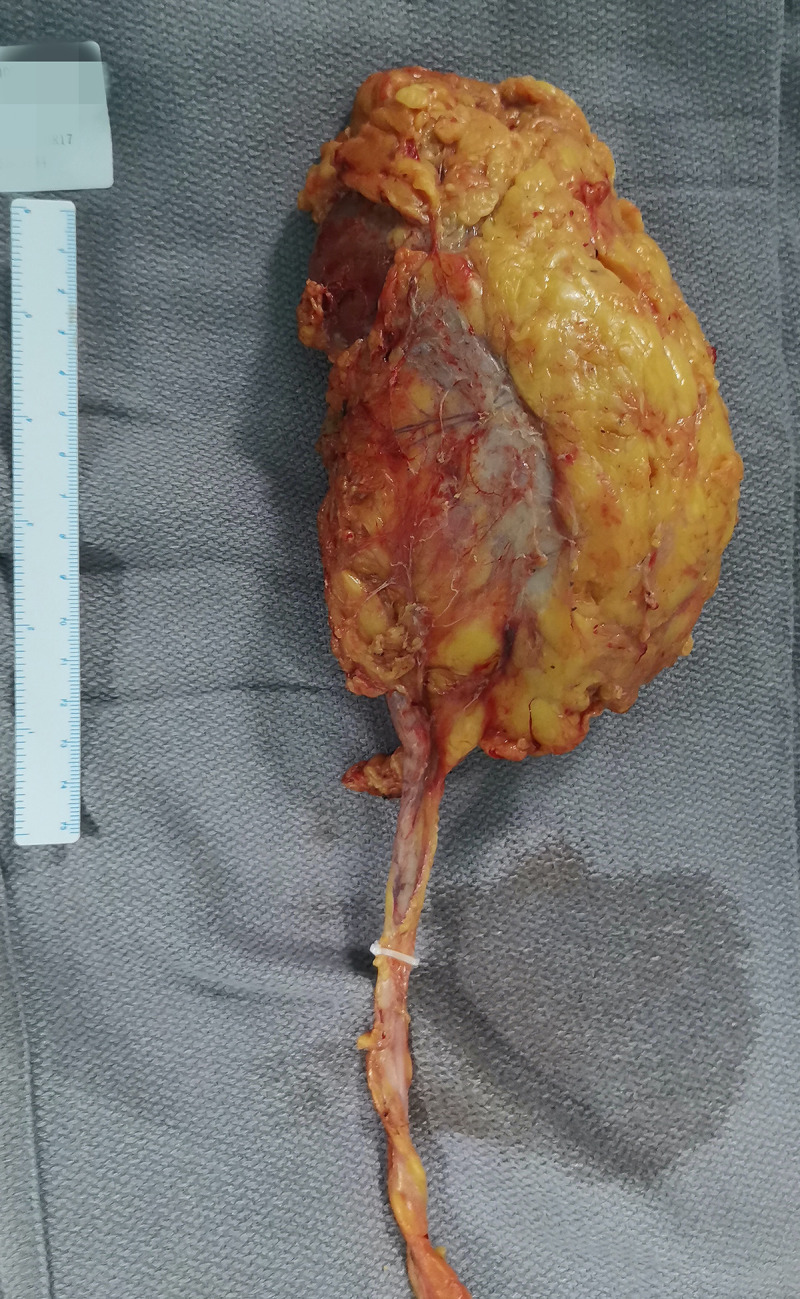
The gross specimen. The right middle ureter was blocked with a Hem-o-lok clip, which avoided the spread and overflow of tumor and pus into the lower urinary tract during the surgery.

**Figure 4:**
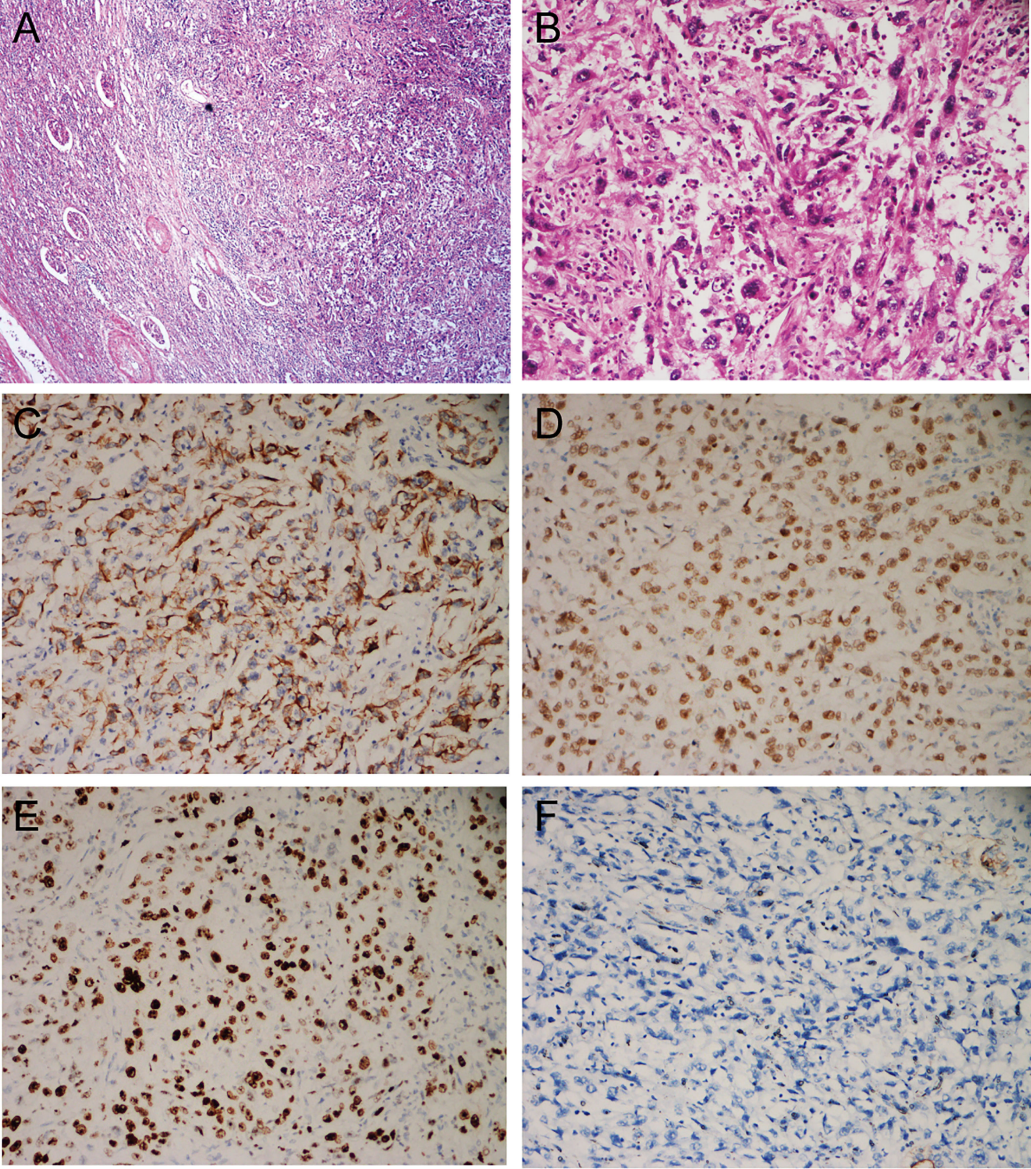
Histological and immunohistochemical features. **(A)** HE, ×40. Microscopically, the boundary of the tumor is poorly defined and invades the renal parenchyma, with remnants of renal tubules and glomeruli visible in the periphery; **(B)** HE, ×200. The main bodies of the tumor cells are arranged in bundles, with obvious necrosis and inflammatory cell infiltration; some of the tumor cells are spindle-shaped, some are square or columnar, with a high degree of heterogeneity, high nucleoplasm ratio, obvious nucleoli, and the presence of giant tumor cells; **(C)** CK7 (+); **(D)** P63 (+); **(E)** GATA3 (+); **(F)** EMA (-).

**Figure 5:**
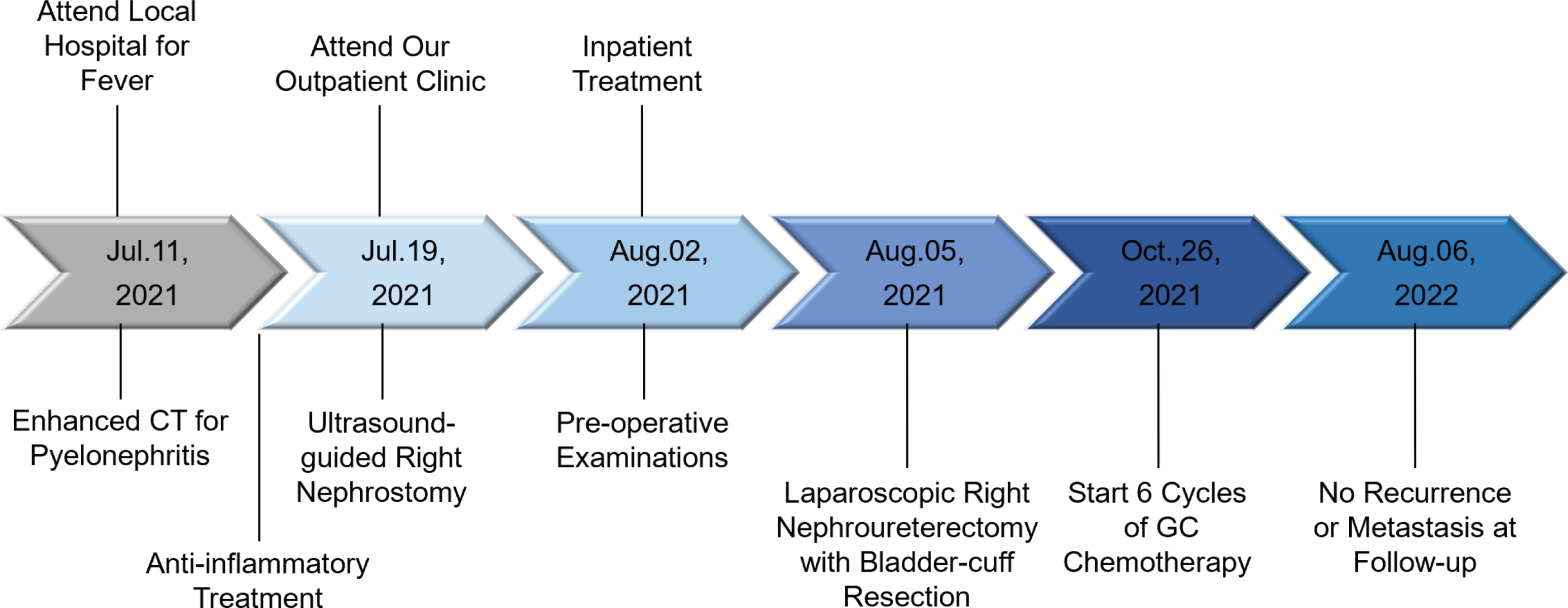
Treatment Timeline.

A 71-year-old male reported the development of a fever of no clear origin that rose to 38.5°C beginning 20 days prior to presentation. The fever was mild in the morning and more pronounced in the evening, and was accompanied by nausea, vomiting, dizziness, right-sided back pain, and frequent urinary urgency. Enhanced computed tomography (CT) imaging revealed abnormalities of the right kidney consistent with potential pyelonephritis. Laboratory test results were as follows: white blood cell count 14.79 x 10^9^ cells/L, urine occult blood (UOB) 1+, urinary leukocytes (U-LEU) 3+, erythrocyte sedimentation rate (ESR) 96 mm/h, urine bacterial culture (-), Widal test (-). The patient did not show any significant improvement after anti-inflammatory treatment, and underwent further evaluation at our hospital. Physical examination indicated the presence of positive percussion pain in the right renal area. Enhanced magnetic resonance imaging (MRI) showed multiple irregular mixed signals in the lower and middle parenchyma of the right kidney, with a long T1 low signal on T1WI and a long T2 high signal on T2WI, with marginal circumferential enhancement on enhancement scans. Diffusion-weighted imaging (DWI) showed a high signal and decreased analog-to-digital converter (ADC) values, with marked diffusion restriction of the pelvic wall and parenchymal lesion margins on both DWI and ADC images. The right renal pelvis and calyces were dilated and the right pelvic wall was thickened, with significant enhancement in the arterial phase seen on enhancement scans. Infectious lesions were first considered and a short review in conjunction with clinical treatment was recommended to exclude neoplastic lesions (**Figure 1** and **Supplementary Figure 1**). Laboratory test results at this time were as follows: WBC 22.89 x 10^9^ cells/L; UOB 3+; U-LEU 3+; estimated glomerular filtration rate (eGFR) 96 mL/min. The results of the urine bacterial culture revealed the presence of enterococcus. The patient did not respond to anti-inflammatory treatment, and underwent ultrasound-guided right nephrostomy, which drained 100 mL of pus and provided significant relief of the back pain. The joint consideration of the imaging findings together with the pus drainage provided proof of the presence of a renal abscess. The laboratory findings on the puncture fluid revealed leukocytes (3+) and acid-fast stain (-). Exfoliative cytology analyses of the puncture fluid were performed, and cancerous cells were detected. Anti-inflammatory treatment was maintained for 10 days, and follow-up CT imaging revealed a nephrostomy drainage tube extending from the right renal pelvis to the outside of the body. The right renal pelvis and part of the calyces were dilated, with thickening and enhancement of the wall. The right kidney was less enhanced than the contralateral side. A mass of slight hypointense lesions, approximately 6.3 x 4.6 cm in cross-section, was seen in the lower and middle parenchyma of the right kidney, with mild to moderate heterogeneous enhancement on enhancement scans. The thickening of the right renal pelvic wall was consistent with an infectious lesion, while the abnormal enhancement of the lower and middle portions of the right kidney was thought to correspond with a tumor, suggested in conjunction with the pathology (**Figure 2** and **Supplementary Figure 2**). Following a discussion with the patient and their family, laparoscopic right nephroureterectomy with bladder-cuff resection was performed in our hospital to avoid any further delay in tumor treatment. The patient was transfused intraoperatively with 2 units of erythrocytes and 400 mL of plasma due to preoperative anemia (hemoglobin: 88 g/L). Twenty-four hours after surgery, the patient received a single dose of intravesical chemotherapy (pirarubicin). The gross specimen is shown in **Figure 3**. Regrettably, we did not photograph the dissected renal pelvis of the specimen, which represents a limitation of our work. The patient recovered well after surgery and was discharged on day 6 postoperatively. Pathology results revealed that the tumor had a biphasic appearance of a high-grade UC and a sarcomatous component with both renal parenchymal and renal sinus fat involvement. Immunohistochemical staining results were P63 (+), CK7 (+), GATA-3 (+), Ki-67+ (60%), P40 (+), epithelial membrane antigen (EMA) (-) and Her2 (0) (**Figure 4**). According to the American Joint Committee on Cancer (AJCC), the pathological stage was determined to be T3NxMx. The renal function was reviewed 50 days after surgery and showed an eGFR of 91 mL/min, with creatinine and urea nitrogen levels both within normal range. The patient received up to six cycles of treatment with gemcitabine and cisplatin (GC). The renal function was assessed during chemotherapy, showing that the patient tolerated chemotherapy with a mild increase in creatinine. Regular follow-up to date has not revealed any evidence of recurrence or metastatic progression for about 1 year. The treatment flowchart is shown in **Figure 5**.

## Discussion

SUC is a rare and highly aggressive tumor type that accounts for 0.3% of all UC cases (1). SUC generally developed in the bladder, and reports of SUCs in the renal pelvis are extremely rare (4), with such descriptions largely confined to individual case reports. The first description of a SUC of the renal pelvis was published by Piscioli et al. in 1984 (5). For a systematic overview of all cases of SUCs of the renal pelvis published after the year 2000, see **Table 1** (1, 2, 4, 6–21).

**Table 1:** Literature review of published cases.

NO.	Author	Age/sex/side	Clinical symptoms	Preoperative diagnosis	Surgical treatment	pathology	Heterologous component	Stage	Adjuvant treatment	Follow-up (months)
1	Vermeulen P, et al.	77/M/R	No	RPT	NU	SC, SCC, ITCC, CIS	Chondrosarcoma	T3N0M0	No	8/NED
2	Hisataki, et al.	43/F/L	Flank pain	MCRCC	N	SC, ITCC	No	NR	No	10/Retroperitoneal recurrence; 14/DOD
3	AcikalinM F, et al.	66/M/L	Hematuria and flank pain	RPT	NU	SC, TCC, SCC	No	T3N1M0	AC(M-VAC)	2/Metastasis(liver, right kidney, thorax); 7/DOD
4	Thiel D D, et al.	61/M/L	Hematuria	RPT	NU	SC, TCC	No	T3N0M0	No	12/NED
5	Masue N, et al.	56/M/L	Hematuria and urinary infection	RPT	NU	SC, TCC	No	T3N0M1	No	5/Disappearance of lung metastases; 46/NED
6	Nakano Y,et al.	81/F/L	Hematuria	RPT	N	SC, PUC	No	TxN0M0	No	1/Metastasis(lung, spine); 2/DOD
7	Gill-Samra S,et al.	76/F/R	Hematuria, anemia and renal insufficiency	RPT	N	SC, SCC, CIS	Osteosarcoma	T2N0M0	No	2/Ureteral stump and bladder recurrence
8	Ahn H I, et al.	68/M/R	Hematuria and flank pain	RPT	NU	SC, PUC	Osteosarcoma	T3N1M0	AC	4/Recurrence in the right subphrenic area and lung metastasis
9	Fernández-Pello S, et al.	58/M/R	Hematuria and septic shock	RA	N	SC, TCC, CIS	No	T4N0M0	No	18/NED
10	Tian X, et al.	49/F/L	Hematuria	RPT	NU	SC, HGTCC	No	T1N0M0	No	30/NED
11	Wang R, et al.	63/M/L	High fever and flank pain	RCC	N	SC, TCC	No	T4N0M0	No	6/Multiple organs metastasis; 10/DOD
12	Lisa M, et al.	47/M/L	Hematuria and flank pain	RI	N	SC, HGTCC, SCC	Osteosarcoma	T4N0Mx	Spine radiotherapy, Sunitinib	1/Bone metastases, surgical site recurrence; 5/DOD
13	Rashid S, et al.	70/M/R	Urgency, urinary incontinence and nocturia	RCC	NU with thrombectomy	SC, HGTCC	No	T3N0M0	NR	NR
14	Mohan B P, et al.	68/F/L	Hematuria	RPT	NU	SC, CIS	Osteosarcoma	T3N0M0	No	10/NED
15	Sekido Y, et al.	72/M/R	Bellyache	RPT	N	SC, TCC, SCC, AC	No	T3N0M0	No	6/NED
16	Hoshiyama A, et al.	75/M/L	General malaise and Hematuria	Pyelonephritis	N	SC, TCC	No	T3NxM0	No	0.33/ Ureteral stump recurrence and liver metastasis; 0.66/DOD
17	Nasrollahi H, et al.	68/F/R	Hematuria and flank pain	RPT	NU	SC, HGTCC	No	T3N0M0	AC(GC), radiotherapy, removal of brain metastases	2/Brain metastases;3/DOD
18	Nakano Y, et al.	75/F/L	No	RCC	N+ removal of transferred organ	SC, ITCC	No	T4N2M1	AC(GP)	1/Metastases(lung, pleural and liver); 5/DOD

M, male; F, female; R, right; L, left; RPT, renal pelvic tumor; MCRCC, multilocular cystic renal cell carcinoma; RA, renal abscess; RCC, renal cell carcinoma; RI, renal insufficiency; NU, nephroureterectomy; N, nephrectomy; SC, sarcomatoid carcinoma; SCC, squamous cell carcinoma; ITCC, invasive transitional cell carcinoma; CIS, carcinoma in situ; TCC, transitional cell carcinoma; PUC, papillary urothelial carcinoma; HGTCC, high grade transitional cell carcinoma; NR, not reported; AC, adjuvant chemotherapy; MVAC, methotrexate+vinblastine+adriamycin+cisplatin; GP, gemcitabine+paraplatin; NED, no evidence of disease; DOD, died of disease.

In total, 19 cases have been reported in individuals ranging from 43-81 years of age (average: 65.7 years), with more cases among males than females (M:F = 12:7). These tumors usually develop on the left side (L:R = 12:7) and are most frequently characterized by hematuria (12 cases) and lumbar pain on the affected side (6 cases), with other symptoms, including general discomfort, high fever, abdominal pain and distension, and urinary tract irritations, being relatively rare. Much as in conventional UC cases, these SUC cases were not associated with any specific symptoms (22). Notably, two patients were asymptomatic and one exhibited invasion of adjacent organs and distant metastases when initially diagnosed (6, 21). In 12 cases, the clinical diagnosis of renal pelvic tumors was confirmed based on a combination of clinical symptoms, imaging findings, and laboratory test results prior to surgery. However, some patients were initially diagnosed with renal tumors (4 cases), pyelonephritis (1 case), renal abscesses (1 case), and renal insufficiency (1 case). In 2014, Fernández-Pello et al. reported a case of a patient with SUC of the renal pelvis that presented as a renal abscess such that the patient experienced hematuria and needed to undergo urgent nephrectomy due to infectious shock (14). The elderly male in the present case initially experienced fever as the first symptom of disease, together with dizziness, nausea, vomiting, back pain on the affected side, and urinary tract irritation. While imaging results led to the consideration of pyelonephritis as a diagnosis in this case, the patient did not respond to anti-inflammatory treatment and his blood leukocyte counts rose further, with repeat imaging suggesting a renal abscess. A nephrostomy procedure enabled the drainage of pus, and exfoliative cytology analyses of the drained fluid were consistent with a possible diagnosis of renal pelvis carcinoma such that radical surgery was performed to avoid a missed diagnosis.

Renal abscesses present as a form of inflammatory disease characterized by back pain, urinary tract irritation, and a high fever (23). These abscesses are most common in young and middle-aged individuals, and primarily result from retrograde infections of the urinary tract or bloodborne infections. Factors related to renal abscess susceptibility include nephrolithiasis, vesicoureteral reflux, and diabetes (24). With respect to the relationship between SUC and renal abscess formation, SUCs are generally relatively large when initially diagnosed, often being up to three times larger than conventional UC tumors (25). As such, the presence of the tumor leading to local obstruction, infection secondary to poor urinary flow and urinary reflux may account for abscess formation. In addition, SUCs are often high-grade highly malignant tumors that grow rapidly, with insufficient neovascularization to supply the tumor with blood, leading to necrosis and the accumulation of dead cells in the obstructed collecting system, thus causing the formation of abscesses (22). Therefore, purulent fluid is sometimes related to the presence of a rapidly growing tumor leading to cell necrosis rather than hematuria. That is also true for plasmacytoid bladder cancer with rapidly growing histological variants (26).

When SUCs of the renal pelvis present in combination with a renal abscess, the abscess often masks the symptoms of the associated tumor, resulting in delayed or missed diagnoses in many cases. As such, it is important that the possibility of neoplastic lesions be considered when evaluating and treating patients with renal abscesses. First, urinary cytology should be performed. It is also useful to analyze DNA methylation sites in urothelial carcinoma or chromosomal instability in the exfoliated cells to facilitate their accurate identification (27). Treatment with anti-inflammatory drugs and pus drainage with a ureteral catheter are then justified. In such cases, cytological analysis of the purulent fluid is mandatory for the detection of viable tumor cells. Repeated imaging can also being used to monitor for any changes in these lesions, with MRI offering a better means of detecting lesion-associated soft tissue while also more readily and sensitively allowing for the detection of necrotic liquefied components (28). Fine-needle aspiration biopsy can be performed in individuals who respond poorly to anti-inflammatory treatment and exhibit suspicious imaging findings. Individuals with the potential for malignant disease must undergo surgical treatment as quickly as possible to avoid further diagnostic or treatment delays.

SUC tumors consist of both mesenchymal and epithelial components of monoclonal origin, and diagnosis is generally dependent on pathological examination (22). Microscopically, conventional urothelial, squamous, glandular, or small-cell components may be mixed with sarcomatous areas composed of spindle cells or pleomorphic cells (14). Heterogenous differentiation may exist in sarcomatoid areas, including osteosarcoma, chondrosarcoma, rhabdomyosarcoma, liposarcoma, and angiosarcoma, which have no clear prognostic significance (22). Sarcomatoid carcinoma and carcinosarcoma appear similar on hematoxylin and eosin (HE) staining, necessitating further immunohistochemical characterization for diagnosis, with the aim of demonstrating uroepithelial or at least epithelial origins (9). The sarcomatoid component can retain at least focal expression of cytokeratins, most often high-molecular weight cytokeratins, and can also express p63 and GATA3 (29).

SUC tumors are extremely aggressive and are significantly associated with advanced pathological T-stage, higher tumor grade, and more frequent regional and distant metastases compared to urothelial carcinoma (30). A study using the National Cancer Institute population based SEER database reported that patients with sarcomatoid carcinoma are at greater risk of death compared to those with urothelial carcinoma, even after adjustment for stage of presentation. In addition, carcinosarcoma carries an increased risk of death compared to sarcomatoid carcinoma, which provides some value to the clinical distinction between the two entities (30). Over a median follow-up period of 6 months (range: 3-46 months), among these 19 patients, 10 patients developed recurrent metastases, of whom 7 exhibited new metastatic lesions within 2 months of surgery, most commonly in the lungs and liver. Only a small number of SUC patients reportedly survive for more than 2 years following diagnosis (22). Accurate tumor staging is critical for efforts to appropriately gauge a given patient’s prognosis (3).

With respect to the treatment of SUC, no specific treatment guidelines have been established, and surgery remains the preferred clinical treatment in published reports (NU:10 cases, N:9 cases). Of patients that underwent nephrectomy, 2 exhibited ureteral stump recurrence within 20 days and 2 months after surgery, and another patient experienced surgical site recurrence 1-month post-surgery. Higher recurrence rates were reported following nephrectomy compared with nephroureterectomy. Current consensus indicates that extensive radical surgery is the most appropriate treatment course in order to ensure negative operative margins, thereby contributing to a better overall prognosis (31). In four reported cases, patients underwent adjuvant chemotherapy treatment with regimens designed to treat conventional uroepithelial carcinoma cases (GC, GP, M-VAC), but all four patients experienced recurrent metastatic disease within 1-4 months post-surgery. In two prior multicenter retrospective studies, the overall survival of SUC patients was not found to improve significantly following chemotherapy treatment (32, 33). Moreover, the eGFR is significantly reduced after nephroureterectomy, particularly in elderly patients. Furthermore, 50% of patients are not eligible for platinum-based chemotherapy due to postoperative renal failure. Therefore, patients are at high risk of postoperatively reduced renal function and it is recommended that neoadjuvant chemotherapy be considered (34). At present, the relative efficacy of adjuvant radiotherapy in SUC cases remains to be established. In two cases, patients were treated with radiation targeted to the sites of the metastatic lesions, but both patients died within a few months of treatment and the beneficial effects of irradiation in these cases are unclear. The advent of novel targeted drugs and immune checkpoint inhibitors has offered new treatment opportunities for advanced UC patients. In a 2009 study, Wang et al. reported that most SUC tumors exhibit moderate-to-strong expression of epidermal growth factor receptor (EGFR), indicating that anti-EGFR regimens may be of value for treating this rare cancer type (35). In a 2022 retrospective analysis of 755 advanced UC patients who underwent pembrolizumab treatment, Kobayashi et al. observed positive responses to treatment and improved survival rates among SUC patients (36). Consistently, one patient with metastatic SUC that failed to benefit from postoperative chemotherapy exhibited significant reductions in the size of metastatic lesions following treatment with the immune checkpoint inhibitor atezolizumab (37). Both targeted and immunotherapeutic regimens exhibit great promise as a treatment for SUC patients, although further large-scale multicenter confirmation of these results is warranted. In the present case, the patient was successfully treated with negative margins, and imaging did not reveal any evidence of lymph node metastases. After 6 cycles of postoperative adjuvant GC chemotherapy and regular follow-up for over 1 year, this patient has not exhibited any evidence of recurrent or metastatic disease.

Despite the good prognosis observed, our work still contains limitations. Firstly, we did not photograph the dissected renal pelvis of the specimen, which limits our understanding of the general appearance of SUC of the renal pelvis. Secondly, the patient underwent right nephrostomy after failure of the anti-inflammatory treatment. Although cancer cells were detected on exfoliative cytology of the puncture fluid, which provided support for definitive surgical treatment as soon as possible, there is a risk of implantation metastasis after nephrostomy. Therefore, it is recommended to drain the pus with a ureteral catheter and perform cytology in patients with renal abscesses when there is suspicion of a tumor on imaging. Thirdly, in this case, lymph node dissection (LND) was not done. Based on the NCCN and EAU clinical practice guidelines, template-based and complete LND improve cancer-specific survival in patients with muscle-invasive disease and reduce the risk of local recurrence (38). Even in node-negative patients, LND improves survival (39). In future clinical practice, we should emphasize the possibility of renal pelvic carcinoma with renal abscess, as this may indicate a poor pathological type with high invasiveness, and standard intraoperative lymph node dissection is necessary for more accurate tumor staging and better prognosis. Besides, the patient was reviewed at the local hospital postoperatively without cystoscopy or urine cytology. The rate of bladder recurrence after RNU for UTUC is 22–47% (40). For high-risk tumors, cystoscopy and urine cytology should be performed every three months. We have suggested that the patient includes a cystoscopy and urine cytology at their next review.

## Conclusion

SUC is a highly aggressive and rare form of uroepithelial malignancy associated with poor prognostic outcomes. Clinicians must be aware of the potential for combined SUC of the renal pelvis in patients with clinical symptoms and imaging findings consistent with a renal abscess. At present, clinical experience in the treatment of such SUC cases is limited, and surgery remains the preferred treatment while targeted and immune therapies offer the potential for further improvements in patient survival. Maintaining negative operative margins and ensuring accurate pathological staging are both critical to accurately gauging a given patient’s prognosis. It is also essential that the delayed or missed diagnosis of such SUC cases be avoided in clinical practice through appropriate vigilance and diagnostic evaluation.

## Data availability statement

The original contributions presented in the study are included in the article/**Supplementary Material**. Further inquiries can be directed to the corresponding author.

## Ethics statement

Written consent for publication of this case report and its accompanying images has been obtained from the patient.

## Author contributions

CY composed the manuscript and literature review. YK performed pathological diagnosis and collected pathological pictures. WD, LF and ZZ collected the clinical data. TC and NH revising it and gave final approval of the version to be published. LJ designed the report. All authors contributed to the article and approved the submitted version.

## Supplementary material

Supplementary Figure 1 Enhanced MRI examination. **(A)**, **E**: axial DWI; **(B)**, **F**: axial ADC imaging; **(C)**: axial T1-weighted arterial phase imaging; **C**: axial T2-weighted imaging.

Supplementary Figure 2 Review of enhanced CT after nephrostomy. **(A)**: excretory phase; **(B)**: arterial phase; **(C)**: venous phase.
