# Supplementary figures and images for: Case report: Sarcomatoid urothelial carcinoma of the renal pelvis masquerading as a renal abscess
Source: Front Oncol. 2023 Jan 23;13:1055229. doi: 10.3389/fonc.2023.1055229 (PMC9899929; doi:10.3389/fonc.2023.1055229)

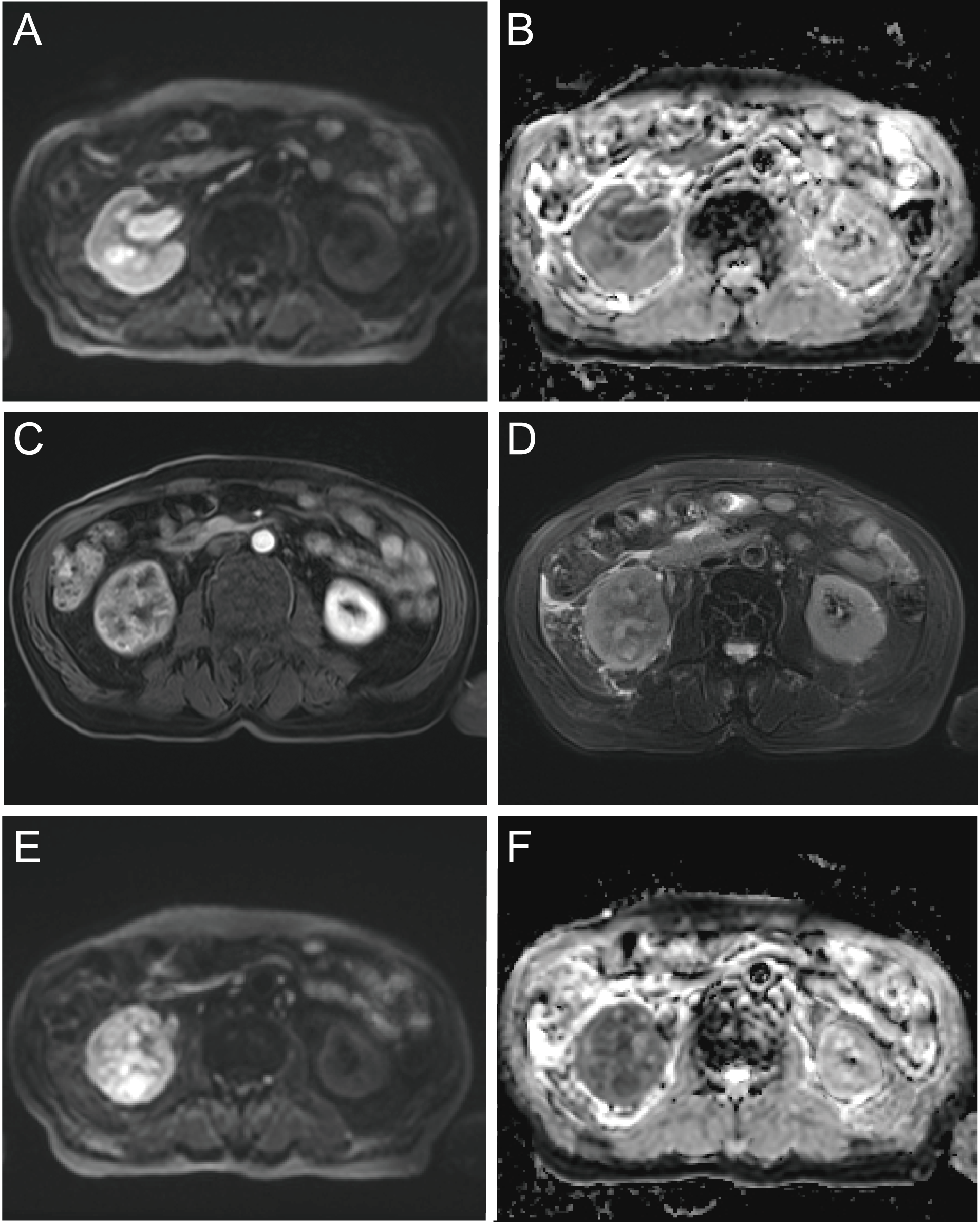

Supplement: Supplementary Figure 1 — Enhanced MRI examination. (A), E: axial DWI; (B), F: axial ADC imaging; (C): axial T1-weighted arterial phase imaging; C: axial T2-weighted imaging. [file Image_1.tif]

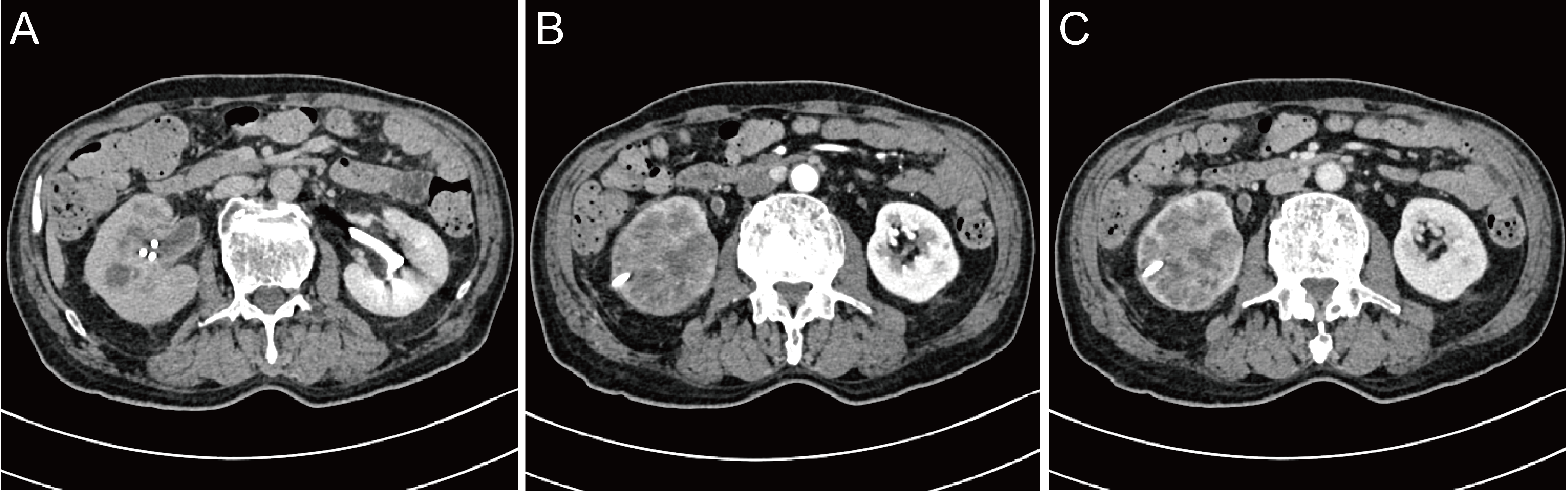

Supplement: Supplementary Figure 2 — Review of enhanced CT after nephrostomy. (A): excretory phase; (B): arterial phase; (C): venous phase. [file Image_2.tif]
